# TRAF3 Is Required for NF-κB Pathway Activation Mediated by HTLV Tax Proteins

**DOI:** 10.3389/fmicb.2019.01302

**Published:** 2019-06-12

**Authors:** Stefania Fochi, Elisa Bergamo, Michela Serena, Simona Mutascio, Chloé Journo, Renaud Mahieux, Vincenzo Ciminale, Umberto Bertazzoni, Donato Zipeto, Maria Grazia Romanelli

**Affiliations:** ^1^Department of Neurosciences, Biomedicine and Movement Sciences, Section of Biology and Genetics, University of Verona, Verona, Italy; ^2^Retroviral Oncogenesis Laboratory, Centre International de Recherche en Infectiologie (CIRI), INSERM U1111 – Université Claude Bernard Lyon 1, CNRS, Equipe Labellisée “Fondation pour la Recherche Médicale”, UMR5308, Ecole Normale Supérieure de Lyon, Université Lyon, Lyon, France; ^3^Department of Surgery, Oncology and Gastroenterology, University of Padua, Padua, Italy; ^4^Veneto Institute of Oncology IOV – IRCCS, Padua, Italy

**Keywords:** HTLV, NF-κB, Tax, HBZ, APH-2, TRAF3

## Abstract

Human T-cell leukemia viruses type 1 (HTLV-1) and type 2 (HTLV-2) share a common genome organization and expression strategy but have distinct pathological properties. HTLV-1 is the etiological agent of Adult T-cell Leukemia (ATL) and of HTLV-1-Associated Myelopathy/Tropical Spastic Paraparesis (HAM/TSP), whereas HTLV-2 does not cause hematological disorders and is only sporadically associated with cases of subacute myelopathy. Both HTLV genomes encode two regulatory proteins that play a pivotal role in pathogenesis: the transactivating Tax-1 and Tax-2 proteins and the antisense proteins HBZ and APH-2, respectively. We recently reported that Tax-1 and Tax-2 form complexes with the TNF-receptor associated factor 3, TRAF3, a negative regulator of the non-canonical NF-κB pathway. The NF-κB pathway is constitutively activated by the Tax proteins, whereas it is inhibited by HBZ and APH-2. The antagonistic effects of Tax and antisense proteins on NF-κB activation have not yet been fully clarified. Here, we investigated the effect of TRAF3 interaction with HTLV regulatory proteins and in particular its consequence on the subcellular distribution of the effector p65/RelA protein. We demonstrated that Tax-1 and Tax-2 efficiency on NF-κB activation is impaired in TRAF3 deficient cells obtained by CRISPR/Cas9 editing. We also found that APH-2 is more effective than HBZ in preventing Tax-dependent NF-κB activation. We further observed that TRAF3 co-localizes with Tax-2 and APH-2 in cytoplasmic complexes together with NF-κB essential modulator NEMO and TAB2, differently from HBZ and TRAF3. These results contribute to untangle the mechanism of NF-κB inhibition by HBZ and APH-2, highlighting the different role of the HTLV-1 and HTLV-2 regulatory proteins in the NF-κB activation.

## Introduction

Human T-cell leukemia virus type 1 (HTLV-1) was the first human retrovirus discovered. It is estimated that 10–20 million people are infected worldwide by this oncogenic virus. The majority of infected carriers remain asymptomatic, but 5–10% of infected subjects are at risk of developing either Adult T-cell Leukemia (ATL), a highly aggressive peripheral T-cell malignancy, or a chronic neurodegenerative disorder called HTLV-1-Associated Myelopathy/Tropical Spastic Paraparesis (HAM/TSP) (Kannian and Green, 2010; Ishitsuka and Tamura, 2014; Bangham et al., 2015). HTLV-1 is genetically related to HTLV type 2 (HTLV-2), which is associated with lymphocyte proliferation and rare cases of subacute myelopathy but does not cause hematological disorders (Araujo and Hall, 2004; Bartman et al., 2008). The difference in the pathobiology of HTLV-1 and HTLV-2 might provide important clues to further understand the mechanisms leading to their distinct clinical outcomes. HTLV-1 and HTLV-2 express the transactivating proteins Tax-1 and Tax-2, respectively, which can immortalize human CD4+ T-cell (Cheng et al., 2012; Ciminale et al., 2014; Ma et al., 2016). Both Tax proteins activate viral RNA transcription from the LTR promoter (Journo et al., 2009), interact and modulate the expression of a wide range of cellular proteins and deregulate multiple cellular pathways (Romanelli et al., 2013). Among them, Tax proteins induce the hyper-activation of NF-κB transcription factors, thus altering the control mechanisms of cell proliferation and survival (Harhaj et al., 2007; Kfoury et al., 2012; Fochi et al., 2018). The mechanism of Tax-mediated NF-κB activation has been intensively studied in the past years showing that Tax-1 interacts with the regulatory IKK-γ subunit of IκB kinase complex, also known as NF-κB essential modulator (NEMO). This interaction results in the constitutive activation of IKKα and IKKβ, that leads to degradation of the inhibitor IκB and activation of the canonical NF-κB pathway (Xiao et al., 2006; Sun, 2017). Several other factors involved in the persistent activation of the NF-κB pathway have been found to interact with Tax-1, including TAB2 (TAK1 binding protein 2), TAX1BP (Tax1 binding protein 1), NRP/Optineurin, TRAF6 and CADM1 (cell adhesion molecule 1) (Avesani et al., 2010; Journo et al., 2013; Pujari et al., 2015). It has been demonstrated that persistent NF-κB activation by Tax-1 induces cell senescence (Kuo and Giam, 2006; Zhi et al., 2011) and this effect may be counteracted by the expression of the HTLV-1 antisense protein HTLV-1 bZIP factor (HBZ). HBZ is an inhibitor of the activation of 5′LTR promoter and of Tax-1-mediated transcription. HBZ is constitutively expressed in most ATL cases and enhances the proliferation of T-cells *in vitro* and *in vivo* (Satou et al., 2006). The combined action of Tax-1 and HBZ is considered relevant for the proliferation of HTLV-1 infected cells and persistent infection (Barbeau et al., 2013; Zhao, 2016). Several studies have demonstrated that HBZ and Tax-1 exert opposite functions in the deregulation of cellular signaling pathways that may help the virus to escape from immune surveillance (Gaudray et al., 2002; Zhao, 2016; Bangham and Matsuoka, 2017; Baratella et al., 2017; Karimi et al., 2017). HBZ selectively inhibits the canonical NF-κB pathway activated by Tax-1 together with the host transcription factor p65, by repressing the p65 ability to bind DNA (Zhao et al., 2009). Furthermore, HBZ reduces p65 acetylation and enhances its degradation through the PDLIM2 E3 ubiquitin ligase, resulting in the reduction of the expression of several NF-κB target genes (Zhao et al., 2009; Wurm et al., 2012). Recently, Ma et al. (2017) have demonstrated that HBZ-mediated NF-κB inhibition contributes to the suppression of cyclin D1 gene expression, favoring the G1/S phase transition of the cell cycle.

HTLV-2 also expresses an antisense transcript, encoding APH-2 (antisense protein of HTLV-2) (Halin et al., 2009), which is widely expressed *in vivo* (Douceron et al., 2012). Unlike Tax-1 and Tax-2, which show a high degree of conservation, APH-2 shows less than 30% similarity to HBZ and does not contain a conventional basic leucine zipper domain. APH-2 is likewise able to inhibit Tax-2-mediated viral transcription by interacting with CREB (Halin et al., 2009; Yin et al., 2012), but its repressive activity is weaker compared to HBZ. It was recently reported that APH-2, like HBZ, represses p65 transactivation. However, APH-2 does not reduce the level of p65 expression nor induces its ubiquitination (Panfil et al., 2016). It is not yet established whether APH-2 inhibits Tax-2-mediated NF-κB activation. Of note, while both Tax-1 and Tax-2 activate the canonical NF-κB pathway, only Tax-1 activates the non-canonical one by recruiting NEMO and IKKα to p100 and promoting the release of p52/RelB active heterodimers into the nucleus (Shoji et al., 2009; Motai et al., 2016). These different mechanisms still need to be adequately addressed. We have recently demonstrated that both Tax-1 and Tax-2 interact with the TNF-receptor associated factor 3 (TRAF3), an adaptor protein that participates in the crosstalk between the type I interferon (IFN-I), the mitogen-activated protein kinase (MAPK) and the NF-κB pathways (Diani et al., 2015). TRAF3 positively regulates IFN-I production, while it inhibits the MAPK pathway and the non-canonical NF-κB pathway (Häcker et al., 2011). TRAF3 is a component of a multiprotein complex containing TRAF2 and the cellular inhibitor of apoptosis proteins cIAP1 and cIAP2, which restrict the activation of the non-canonical NF-κB pathway. TRAF3 also participates in the degradation of the alternative NF-κB inducing kinase NIK (Hauer et al., 2005; Vallabhapurapu et al., 2008; Zarnegar et al., 2008; Hildebrand et al., 2011), acting as a negative regulator of the non-canonical NF-κB pathway (Yang and Sun, 2015). The accumulation of NIK leads to IKKα activation and p100 processing to yield p52 (Sun, 2017). We have also demonstrated that the IFN-β promoter activation is increased when Tax-1 and TRAF3 are co-expressed with IKK𝜀 or TBK1 (Diani et al., 2015). The impact of TRAF3 on HTLV-mediated NF-κB activation has not yet been understood. In the present study, we demonstrate that TRAF3 plays a critical role in Tax-mediated NF-κB activation. We further show that APH-2, unlike HBZ, may form complexes with Tax-2 and key factors of the NF-κB signaling pathway, decreasing p65 nuclear translocation. These results can contribute to highlight a novel regulatory mechanism of NF-κB activation mediated by HTLV proteins.

## Materials and Methods

### Cell Lines and Transfection

HeLa, HEK293T, TRAF3 knock-out (TRAF3-KO) and U2OS cells were maintained in Dulbecco’s modified Eagle’s Medium (DMEM) supplemented with 10% fetal calf serum (FCS), L-glutamine (2 mM), and Penicillin G (100 U/L)/Streptomycin (100 mcg/L). Jurkat T-cells (clone E6-1) were grown to a density of 5 × 10^5^ cells/mL in RPMI-1640 medium supplemented with 10% FCS, L-glutamine (2 mM) and Penicillin G (100 U/L)/Streptomycin (100 mcg/L). All cell lines were grown at 37°C in a humidified atmosphere with 5% CO_2_. For immunoprecipitation and confocal analysis, 4 × 10^5^ HEK293T and HeLa cells were seeded in 6-well plates. For transactivation studies, 2 × 10^5^ HEK293T cells were seeded in 12-well plates and transfected using TransIT^®^-LT1 transfection reagent (MIR2300, Mirus Bio), following the manufacturer’s protocol. For confocal and transactivation analysis of Jurkat cells, 2 × 10^6^ cells were transfected by electroporation using the Neon Transfection System (Thermo Fisher Scientific), applying three pulsations of 10 ms at 1,325 V.

### CRISPR/Cas9 Knockout of TRAF3

To induce the TRAF3 knockout, two guide RNA (gRNA) sequences 5′-AGCCCGAAGCAGACCGAGTG-3′ and 5′-TCTTGACACGCTGTACATTT-3′ were designed to target exon 1 and exon 2 of the TRAF3 gene, respectively. gRNAs were selected using online tools^1^ (Ran et al., 2013). A third gRNA sequence, 5′-CCAGTTTTTGTCCCTGAACA-3′ was selected to target exon 1 of TRAF3 gene (Chen et al., 2015). Potential off-target sites were predicted using the online tool “CHOPCHOP”^2^ (Labun et al., 2016). Each selected gRNA was cloned independently, using the T4 DNA ligase (Promega) into the BbsI restriction sites of the pSpCas9(BB)-2A-Puro (PX459) V2.0 vector (#62988, Addgene) and transfected in HEK293T cells using the TransIT^®^-LT1 transfection reagent (MIR2300, Mirus Bio), following the manufacturer’s protocol. Cells were selected using 0.5 μg/ml puromycin for 3 days after transfection. Clonal cell lines were isolated by limiting dilution and the absence of the TRAF3 protein was tested by western blot using an anti-TRAF3 specific antibody.

### Plasmids

pJFE-Tax-1, pJFE-Tax-2B full length expression vectors and the plasmid constructs expressing Tax-M22 and Tax-1 K1-10R mutants have been previously described (Turci et al., 2006, 2012). pFlag-APH-2, pGFP-APH-2, pFlag-HBZ, expression plasmids were kindly provided by Dr. Sheehy (Marban et al., 2012). pRSV-RelA/p65, pCMVF-TAB2 and Flag-TRAF3, HA-TRAF3 expression vectors have been previously described (Avesani et al., 2010; Diani et al., 2015). pEF-p52 was kindly provided by Dr. Matsuoka. pcDNA3-VSV-APH-2, pSG5M-Tax2-His, His-HBZ have been previously described (Journo et al., 2013; Dubuisson et al., 2018). pSpCas9(BB)-2A-Puro (PX459) V2.0 vector was purchased from Addgene. NF-κB-Luc and phRG-TK plasmids have been previously described (Bergamo et al., 2017).

### Antibodies

The following primary antibodies were used: mouse monoclonal anti-Flag M2 (F3165, Sigma-Aldrich), rabbit polyclonal anti-Flag (F7425, Sigma-Aldrich), mouse monoclonal anti-VSV (V5507, Sigma-Aldrich), rabbit polyclonal anti-VSV (V4888, Sigma-Aldrich), goat polyclonal anti-His (ab9136, Abcam), rabbit polyclonal anti-HA (H6908, Sigma-Aldrich), mouse monoclonal anti-HA (16B12, Covance), rabbit polyclonal anti IκB-α (#9242, Cell Signaling), rabbit polyclonal anti-TRAF3 (18099-1-AP, ProteinTech), mouse monoclonal anti-p65 (C-20) (sc-372, Santa Cruz), rabbit polyclonal anti-p65 (MAB3026, Merck Millipore), mouse monoclonal anti-IKKγ (611306, BD Bioscience), rabbit monoclonal anti-NF-κB2 p100/p52 (#3017, Cell Signaling Technology), mouse monoclonal anti-β-tubulin (bsm-33034M, Bioss Antibodies), mouse monoclonal anti-Tax-1 derives from hybridoma 168-A51 (AIDS research and Reagent Program, National Institutes of Health), rabbit polyclonal anti-Tax-2 has been previously described (Turci et al., 2012). Horseradish peroxidase-conjugated secondary antibodies anti-mouse IgG (31430, Thermo Fisher Scientific) and anti-rabbit IgG (31460, Thermo Fisher Scientific) were used in western blotting. The secondary antibodies anti-rabbit488 (ab98488, Abcam), anti-goat488 (ab150129, Abcam), anti-mouse549 (DI-2549, Vector Lab), anti-goat TexasRed (705-075-147, Jackson Imm. Res.), (ab150107, Abcam), anti-rabbit649 (STAR36D649, AbD serotec) were used in immunofluorescence.

### Co-immunoprecipitations

HEK293T cells were harvested 24 h after transfection with 1 μg of each expression vector. Cells were lysed in non-denaturing buffer (10 mM Tris–HCl pH 7.5, 5 mM EDTA, 150 mM NaCl, 1% TritonX-100) supplemented with protease inhibitors Complete Protease Inhibitor Cocktail EDTA-free (Roche). Lysates were sonicated twice for 5 s, frozen at -80°C for 1 h and then centrifuged for 30 min at 14000 rpm at 4°C. Proteins were subjected to co-immunoprecipitation with the appropriate primary antibodies overnight at 4°C. Immunocomplexes were linked to magnetic beads of Dynabeads Protein G or A (LifeTechnologies) for 30 min at 4°C. The beads were then washed 3 times and resuspended in elution buffer containing NuPAGE loading buffer (LifeTechnologies) and 0.25 mM DTT.

### Western Blotting

Total protein concentration in cell lysates was determined by Bradford Coomassie brilliant blue assay (Sigma-Aldrich). Equal amounts of cellular proteins were resolved in SDS polyacrylamide gel electrophoresis (SDS-PAGE) and transferred to a PVDF membrane (GE Healthcare). Membranes were first saturated in TBS solution containing 5% non-fat milk and 0.1% Tween20, and then incubated with specific primary and secondary antibodies. Anti-β-tubulin was used as loading control. Bound antibodies were revealed using ECL prime western blotting detection reagent (GE Healthcare), according to the manufacturer’s instructions. Densitometry analysis of western blot protein bands was performed using the GelQuant. NET software provided by http://biochemlabsolutions.com.

### Luciferase Reporter Assay

Luciferase functional quantitative assay was performed as previously described (Bergamo et al., 2017). Briefly, HEK293T, TRAF3-KO and Jurkat cells were transfected with 500 ng of the NF-κB-Luc reporter plasmid together with the appropriate expression vectors or an empty vector. Transfection efficiency was normalized using 100 ng of phRG-TK plasmid (*Renilla* luciferase vector). TNF-α-induced NF-κB-activation was analyzed following transient expression of a NF-κB-luciferase reporter vector and cell stimulation with 25 ng/mL TNF-α for 18 h. Luciferase activity was assayed 24 h post-transfection using the Dual-Luciferase reporter assay system (Promega). Levels of Firefly and Renilla luciferase were measured with a 20/20n Single Tube Luminometer (Promega). Experiments were repeated at least three times and luciferase activity was measured after deduction of the activity levels with the promoter alone.

### Fluorescence Microscopy

HeLa, HEK293T, TRAF3-KO, U2OS and Jurkat cells were transfected with 1 or 2 μg of each appropriate expression vector; p65 protein nuclear translocation was analyzed following 25 ng/mL TNF-α cell stimulation for 20 min. Cells were seeded on cover glasses and 24 h post-transfection HeLa, HEK293T, TRAF3-KO, and U2OS cells were fixed with 4% paraformaldehyde/PBS, whereas Jurkat cells were fixed with formalin (HT5011, Sigma-Aldrich), for 20 min. Cells were then permeabilized with 0.5% Triton X-100/PBS for 20 min, blocked with a 5% milk/PBS solution and incubated with appropriate primary and conjugated secondary antibodies. The cover glasses were then mounted in DAPI-containing Fluoromount-G (Southern Biotech). For confocal analyses, slides were examined under a LSM800 (Carl Zeiss MicroImaging) confocal microscope equipped with 63× 1.4 plan apochromat oil-immersion objective using the ZEN software. For epifluorescence imaging, slides were examined under an AxioImager.Z1 microscope (Zeiss) using the Methamorph software. To quantify p65 nuclear translocation, the ratios of the nuclear/total p65 mean brightness staining were calculated using ImageJ software. RGB profiles were calculated using the RGB profiler plugin.

### RNA Isolation and Quantitative Real-Time PCR

Total RNA was extracted from HEK293T and TRAF3-KO cells using TRIzol^TM^ (Invitrogen), according to the manufacturers’ recommendations. RNA was quantified by NanoDrop 2000 Spectrophotometer (Thermo Fisher Scientific) and absorbance ratio at 260/280 and 260/230 were measured. Total RNA (1 μg) was reverse transcribed at 50°C for 45 min using oligo dT primers and the SuperScript^TM^ III First-Strand Synthesis System (Invitrogen).

PCR reactions were performed using the Power SYBR^®^ Green PCR Master Mix (Applied Biosystems) and ran on CFX Connect^TM^ Real-Time Detection System (Bio-Rad). Primer pairs for TNF Alpha Induced Protein 3 (TNFAIP3/A20), Apoptosis Regulator Bcl-2 (BCL2), Interleukin 6 (IL-6), and Ribosomal Protein Lateral Stalk Subunit P0 (RPLP0) gene expression analysis are listed in Table 1. PCR cycles included an initial step of 10 min at 95°C required for enzyme activation, followed by 40 cycles of amplification: denaturation for 15 s at 95°C, annealing and extension for 60 s at 60°C. The relative fold change values were calculated using the 2^-ΔΔCt^ method (Livak and Schmittgen, 2001). RPLP0 was used as internal reference gene.

**Table 1:** qRT-PCR primer sequences.

Gene	Accession no.	Primer sequence (5′ -≥ 3′)	PCR product (pb)
*TNFAIP3 (A20)*	NM_001270507.1	F: CTGGGACCATGGCACAACTC R: CGGAAGGTTCCATGGGATTC	182
*BCL2*	NM_000633.2	F: GATGTGATGCCTCTGCGAAG R: CATGCTGATGTCTCTGGAATCT	92
*IL6*	NM_000600.4	F: TACATCCTCGACGGCATCTC R: TGGCTTGTTCCTCACTACTCT	247
*RPLP0*	NM_001002.4	F: ACATGTTGCTGGCCAATAAGGT R: CCTAAAGCCTGGAAAAAGGAGG	127

### Statistical Analyses

All data are presented as the means ± standard deviation from at least three independent experiments. Statistical significance was assessed by the Student’s *t*-test. Differences were considered to be significant when *P* < 0.05 (^∗^), and strongly significant when *P* < 0.01 (^∗∗^), and *P* < 0.001 (^∗∗∗^).

## Results

### TRAF3 Is Required for an Efficient Activation of NF-κB Mediated by Tax

**FIGURE 1:**
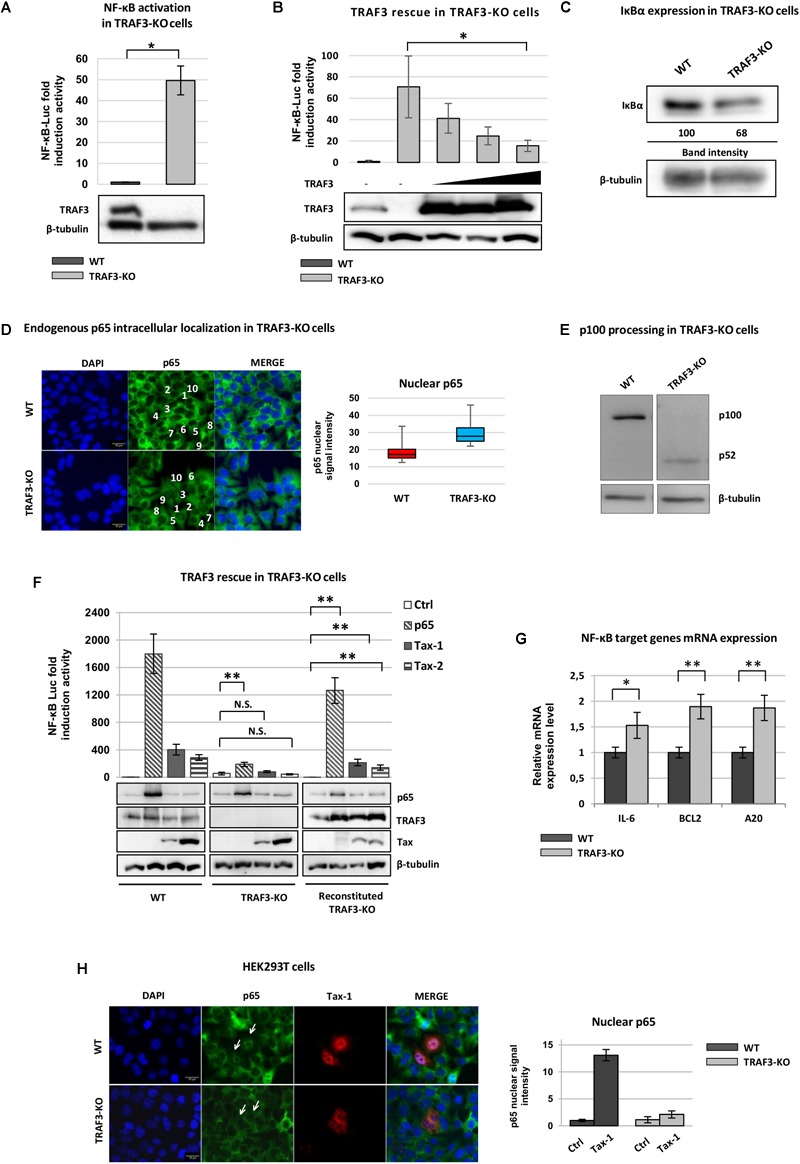
TRAF3 is required for an efficient activation of NF-κB mediated by Tax. **(A)** Luciferase levels were measured from HEK293T (WT) and TRAF3-KO cells transfected with phRG-TK renilla vector (control for transfection efficiency) and NF-κB-luciferase reporter expressing vector. **(B)** Luciferase levels were measured from HEK293T (WT) and TRAF3-KO cells transfected with phRG-TK renilla vector, NF-κB-luciferase reporter and increasing amount of Flag-TRAF3 expressing vector. **(C)** HEK293T (WT) and TRAF3-KO cells were lysed and immunoblot analysis was performed to compare the levels of endogenous IκB. The amount of IκB was measured relative to the amount of β-tubulin. **(D)** HEK293T (WT) and TRAF3-KO cells were stained with anti-p65 (green signal) primary antibody. Nuclei were stained with DAPI (blue signal). Scalebar, 10 μm. Total cells and nuclei of cells (*n* = 10) indicated by a number from 1 to 10 in white were delineated using ImageJ software, and the mean brightness ratio of the nuclear RelA/p65 staining was calculated and presented as box-plot. **(E)** Immunoblot analysis of p100/p52 proteins expression levels in HEK293T (WT) and TRAF3-KO cells. **(F)** HEK293T (WT) and TRAF3-KO cells were transfected with phRG-TK renilla vector, NF-κB-luciferase reporter and p65, Tax-1, Tax-2, or TRAF3 expression plasmids. Cell lysates were collected and luciferase levels were measured. Immunoblot analysis was performed to detect of TRAF3, p65, Tax-1 and Tax-2 (N.S. = not significant). **(G)** RNA from HEK293T (WT) and TRAF3-KO cells were analyzed by qRT-PCR for NF-κB-related transcripts. The values were normalized to RPLP0 and the results were expressed as relative fold change of expression levels. **(H)** HEK293T (WT) and TRAF3-KO cells were transfected with Tax-1 encoding vector and stained with anti-p65 (green signal) and anti-Tax-1 (red signal) primary antibody. Nuclei were stained with DAPI (blue signal). Total cells and nuclei were delineated using ImageJ software, and the mean brightness ratio of the nuclear p65 staining was calculated (right panel). Scalebar, 10 μm. *P* < 0.05 (^∗^) and *P* < 0.01 (^∗∗^).

It has been shown that ATL cells exhibit high expression levels of NIK, a positive regulator of the NF-κB pathway and that NIK turnover is regulated by protein complexes that contain the E3 ubiquitin ligase TRAF3 (Chan and Greene, 2012). Based on our previous data showing that TRAF3 interacts with both Tax-1 and Tax-2 (Diani et al., 2015), we investigated the contribution of TRAF3 in the modulation of NF-κB mediated by the viral regulatory proteins. We produced TRAF3-KO HEK293T cell lines introducing insertion/deletion (**indel**) mutations by transfection of TRAF3 RNA-guided Cas9 nuclease expressing plasmid. TRAF3-KO clones were first characterized for their ability to activate an NF-κB promoter. Using the luciferase reporter assay, we first found that in the absence of any stimulus, the basal NF-κB promoter activity was increased 50 folds in TRAF3-KO cell lines as compared to wild type cells (WT) (Figure 1A). The luciferase activity was restored by the rescue of TRAF3 expression in transfected TRAF3-KO cells (Figure 1B). The basal activation of NF-κB in TRAF3-KO cells was further confirmed by analyzing IκB protein level. As expected, we measured a reduced amount of IκB (30% decrease) in TRAF3-KO cells as compared to WT cells (Figure 1C). When the basal endogenous p65 distribution was analyzed in TRAF3-KO cells, the presence of p65 in the nucleus, even if limited to a weak signal, was observed (Figure 1D). The mean brightness ratio of the nuclear p65 staining was increased 1.5 fold in the TRAF3-KO cells compared to the WT. This indicates that the canonical NF-κB pathway is constitutively although partially activated in the absence of TRAF3. To define if the alternative NF-κB pathway is also activated in the absence of TRAF3, the processing of the p100 protein was analyzed. As shown in Figure 1E, accumulation of p52 was detectable in the TRAF3-KO cell line, but not in the WT cells. These results showing that the depletion of TRAF3 enables detectable translocation of p65 into the nucleus, as well as p100 processing, suggest that both the canonical and non-canonical NF-κB may be partially activated.

When we analyzed the NF-κB activation induced by the expression of p65 in TRAF3-KO cells, we unexpectedly measured a limited induction compared to WT cells (about 9-fold decrease). A similar dramatic reduction of NF-κB induction by Tax-1 or Tax-2 was observed in TRAF3-KO cells compared to WT cells (about 5-fold and 6-fold decrease, respectively) (Figure 1F). Reconstituted TRAF3 expression restored the efficiency of p65- and Tax-mediated activation. In order to explore the mechanism that impairs the full activation of NF-κB mediated by p65 in the absence of TRAF3, we analyzed TNF-α-mediated induction of the NF-κB promoter in TRAF3-KO cells. After TNF-α stimulation, we measured a significant NF-κB activation in TRAF3-KO cells, although lower when compared to WT cells (Supplementary Figure 1A). When we analyzed p65 nuclear translocation induced by TNF-α, we observed p65 nuclear translocation in WT and TRAF3-KO cells (Supplementary Figure 1B), indicating that the NF-κB pathway was activated, although the resulting transcriptional activity of p65 was reduced.

To explore the possibility that in TRAF3-KO cells the basal activation of NF-κB may lead to the expression of NF-κB target genes that control p65 activity by negative feedback loops, we analyzed the expression of A20 and BCL2 genes, which are well known negative regulators of NF-κB (Grimm et al., 1996; Pujari et al., 2013). We found that both genes were significantly more expressed in TRAF3-KO cells compared to WT suggesting that limited p65-mediated induction of NF-κB in TRAF3-KO cells can be due to negative feedback mechanisms (Figure 1G). Furthermore, the results showed an increased expression of IL-6, an NF-κB target gene (Libermann and Baltimore, 1990) (Figure 1G) compared to the WT cells.

In agreement with the results showing that Tax-1 mediated NF-κB activation is impaired in TRAF3-KO cells, we observed that Tax-1 failed to induce endogenous p65 nuclear translocation in transfected cells (Figure 1H). Taken together, these results strongly suggest that NF-κB hyper-activation by Tax is strictly dependent upon TRAF3 expression.

### HBZ and APH-2 Interact With p65 and Suppress the NF-κB Pathway

Since our results indicate that TRAF3 is involved in Tax-induced NF-κB hyper-activation, and it is known that HBZ and APH-2 interfere with NF-κB signaling, we aimed to analyze whether the antisense proteins might interfere with TRAF3 functions. Before analyzing the specific interplay of HBZ and APH-2 with TRAF3, we first aimed at precisely comparing their ability of antagonize Tax-induced NF-κB activation.

)

**FIGURE 2:**
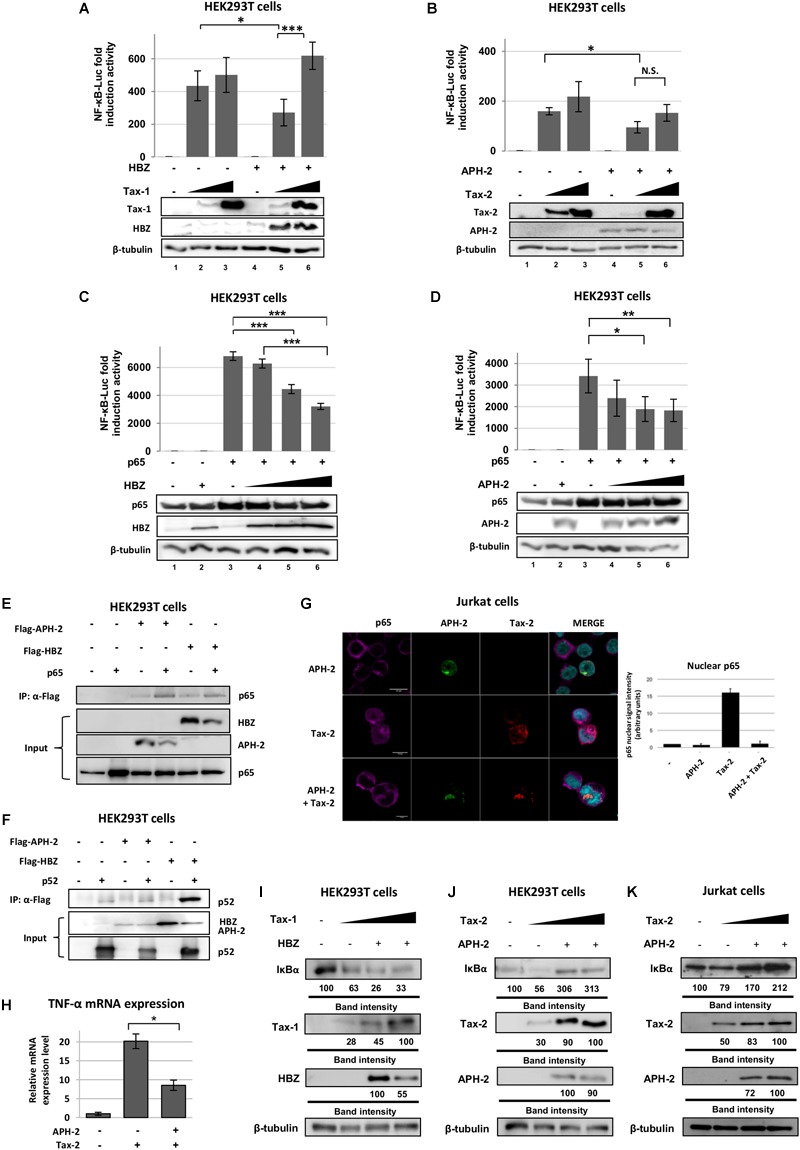
HBZ and APH-2 interact with p65 and suppress the NF-κB pathway. HEK293T cells were transfected with phRG-TK renilla vector, NF-κB-luciferase reporter, Flag-HBZ and increasing amounts of Tax-1 **(A)** or Flag-APH-2 and increasing amounts of Tax-2 expression plasmids **(B)** (N.S. = not significant). HEK293T cells were transfected with phRG-TK renilla vector, NF-κB-luciferase reporter, p65 expression plasmid, and increasing amounts of Flag-HBZ **(C)** or Flag-APH-2 **(D)** expression vectors. Cell lysates were collected and luciferase levels were measured (Top). Immunoblot analysis was performed to detect the expression levels of the transfected proteins (Bottom). **(E,F)** HEK293T cells were transfected with p65 or p52, Flag-APH-2, or Flag-HBZ expression vectors. Tagged proteins were immunoprecipitated with an anti-Flag antibody and the presence of the p65 or p52 proteins was examined by western blot. **(G)** Jurkat cells were transfected with VSV-APH-2 and Tax-2 expression vectors. Samples were stained with anti-VSV, anti-Tax-2 and anti-p65 primary antibodies to detect APH-2 (green signal), Tax-2 (red signal), and endogenous p65 (magenta signal), respectively. Total cells and nuclei were delineated using ImageJ software, and the mean brightness ratio of the nuclear p65 staining was calculated (right panel). Values obtained in non-transfected cells were then subtracted from those for APH-2, Tax or APH-2+Tax-expressing cells. Scalebar, 10 μm. **(H)** TNF-α expression was analyzed by qRT-PCR from RNA of HEK293T cells transfected with VSV-APH-2 and Tax-2. The values were normalized to RPLP0 and the results were expressed as relative fold change of expression levels. **(I–K)** HEK293T and Jurkat cells were co-transfected with Flag-HBZ and increasing amount of Tax-1 or with Flag-APH-2 and increasing amount of Tax-2. Immunoblot analysis was performed to compare the levels of endogenous IκB in the presence of the viral regulatory proteins. The amount of IκB was measured relative to the amount of β-tubulin. *P* < 0.05 (^∗^), *P* < 0.01 (^∗∗^), and *P* < 0.001 (^∗∗∗^).

HEK293T cells transfected with increasing amounts of Tax proteins in the presence or absence of HBZ or APH-2 were analyzed by luciferase assay (Figure 2A,B). As expected, when HBZ was present together with low levels of Tax-1, the NF-κB activation was significantly reduced (Figure 2A, lanes 2 vs. 5), whereas no HBZ inhibitory effect was evident when Tax-1 was expressed at high levels in transfected cells (Figure 2A, lane 3 vs. 6). APH-2 effect on Tax-mediated NF-κB activation was then analyzed (Figure 2B). Similar to HBZ, APH-2 inhibited Tax-2-induced NF-κB activity, but unexpectedly and unlike Tax-1, high expression of Tax-2 did not restore the activation of NF-κB (Figure 2B). Of note, this inhibitory effect of APH-2 was evident despite the fact that APH-2 was less expressed than HBZ in transfected cells, as previously reported (Panfil et al., 2016; Dubuisson et al., 2018). Because APH-2 appeared to be more potent than HBZ in the inhibition of Tax-mediated NF-κB activation, the effect of HBZ and APH-2 expression on the NF-κB promoter activation mediated by p65 over-expression was then compared in HEK293T cells. The inhibition was statistically significant for both proteins and reached similar levels (Figure 2C,D). The results were also reproduced in Jurkat cells in the presence of APH-2 (Supplementary Figure 2A).

To further compare the mechanism of NF-κB inhibition by both antisense proteins, we then analyzed the interaction of HBZ and APH-2 with the transcription factors p65 and p52 by immunoprecipitation. Figure 2E shows that p65 was detected in complexes with both proteins, indicating that both antisense proteins interact with the classical transcription factor p65. When HBZ and APH-2 interaction with p52 was analyzed, it revealed that HBZ was present in complex with p52, unlike APH-2 (Figure 2F). These results suggested that HBZ and APH-2 share the ability to form complexes with p65, but they differ in their interaction with p52, the final effector of the alternative NF-κB signaling. Given that p65 translocates into the nucleus when Tax is expressed, the cellular localization of p65 in the presence of APH-2 and/or Tax-2 was analyzed. The results showed that endogenous p65 localized in the cytoplasm in the presence of APH-2 (Figure 2G). Interestingly, it was found that the co-expression of APH-2 and Tax-2 resulted in their cytoplasmic co-localization and in the impairment of p65 nuclear translocation (5-fold decrease) (Figure 2G). The same results were observed in U2OS cells (Supplementary Figure 2B). We performed qRT-PCR analysis in the presence of Tax-2 and APH-2, analyzing the expression of TNF-α as an example of an NF-κB target gene. In the presence of APH-2 and Tax-2, TNF-α expression was reduced more than 10-fold (Figure 2H). We then analyzed the effect of HBZ and APH-2 on IκB degradation, which allows p65 nuclear translocation and which is known to be induced by Tax (Harhaj and Harhaj, 2005). As expected, the expression of IκB was reduced to about 40% in the presence of Tax-1 and 80% in the presence of both Tax-1 and HBZ proteins (Figure 2I), whereas in the presence of both APH-2 and Tax-2 proteins, an increase in IκB expression was observed (Figure 2J). A similar result was obtained in Jurkat cells (Figure 2K). Taken together, these results suggest that while HBZ and APH-2 both inhibit Tax- and p65-mediated NF-κB activation, they may differ in their inhibition mechanism. APH-2 may inhibit p65 translocation by affecting IκB degradation, thus limiting Tax-2-mediated NF-κB activation.

### APH-2 Is Recruited to Cytoplasmic Structures in the Presence of Tax-2

**FIGURE 3:**
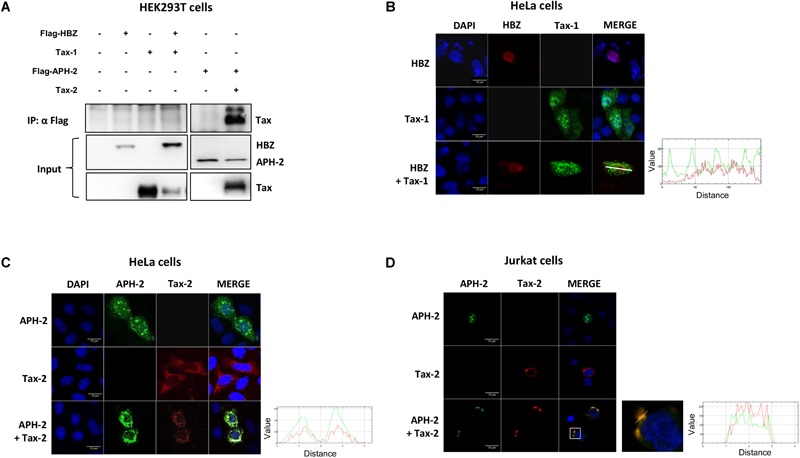
APH-2 is recruited to cytoplasmic structures in the presence of Tax-2. **(A)** HEK293T cells were transfected with Tax-1 and Flag-HBZ or Tax-2 and Flag-APH-2 expression vectors. Tagged proteins were immunoprecipitated with an anti-Flag antibody and the presence of Tax protein was examined by western blot. **(B,C)** HeLa cells were transfected with Tax-1 and Flag-HBZ or Tax-2 and GFP-APH-2 expression plasmids. Cells were stained with anti-Flag and anti-Tax-1 primary antibodies to detect HBZ (red signal) and Tax-1 (green signal), respectively; anti-Tax-2 primary antibody was used to detect Tax-2 (red signal) whereas APH-2 is fused to a GFP protein (green signal). **(D)** Jurkat cells were transfected with VSV-APH-2 and Tax-2 vectors. Cells were stained with anti-VSV, anti-Tax-2 primary antibodies to detect APH-2 (green signal) and Tax-2 (red signal), respectively. Co-localization of Tax-2 signal with APH-2 signal was calculated using Mander’s coefficient (M_1_= 0.91; M_2_= 0.76). Nuclei were stained with DAPI (blue signal). Enlargements are shown next to the “Merge” panel. The intensity of fluorescence along the white line drawn on the merged images is plotted in the diagrams. Scalebar, 10 μm.

To investigate how APH-2 might inhibit NF-κB activity upstream of IκB degradation, we analyzed APH-2 subcellular localization. Indeed, it has previously been reported that while Tax-1 does not interact with HBZ, Tax-2 does interact with APH-2 (Zhao et al., 2009; Marban et al., 2012). Given the different cellular distribution of Tax-1 and Tax-2 (Meertens et al., 2004; Bertazzoni et al., 2011), it is possible that HBZ and APH-2 differ in the mechanism of NF-κB inhibition due to their differential recruitment by host factors in subcellular compartments. To test this hypothesis, HEK293T cells were transfected with Tax-1 or Tax-2 and Flag-HBZ or Flag-APH-2 expressing vectors, and the presence of Tax in the immunocomplexes precipitated with an anti-Flag antibody was analyzed (Figure 3A). The results, as expected, confirmed that Tax-2 formed complexes with APH-2, but no interaction of Tax-1 with HBZ was observed. Confocal microscopy analyses in transfected HeLa cells showed that HBZ alone localized in the nucleus and that when Tax-1 was co-expressed with HBZ, no co-localization was observed (Figure 3B). Interestingly, the co-expression of APH-2 and Tax-2 resulted in APH-2 redistribution and its co-localization with Tax-2 in cytoplasmic structures (Figure 3C). This result was also reproduced in Jurkat cells (Figure 3D). To ensure that recruitment of APH-2 in cytoplasmic structures is a property of Tax-2 but not of Tax-1, HEK293T cells were transfected with both APH-2 and Tax-1, or with both HBZ and Tax-2 vectors and their intracellular localization was analyzed by fluorescent microscopy. We observed that Tax-1 and APH-2 did not co-localize in the cytoplasm (Supplementary Figure 3A). In the presence of HBZ, Tax-2 was mainly distributed in the cytoplasm and HBZ in the nucleus (Supplementary Figure 3B). Taken together, these results suggest that HBZ and APH-2 differ in their subcellular distribution when Tax proteins are expressed and that the cytoplasmic co-localization occurs only in the presence of Tax-2 and APH-2. This might explain how HBZ and APH-2 differ in the mechanism of NF-κB inhibition.

### APH-2 Is Present in Cytoplasmic Complexes With TAB2, NEMO, and Tax-2

**FIGURE 4:**
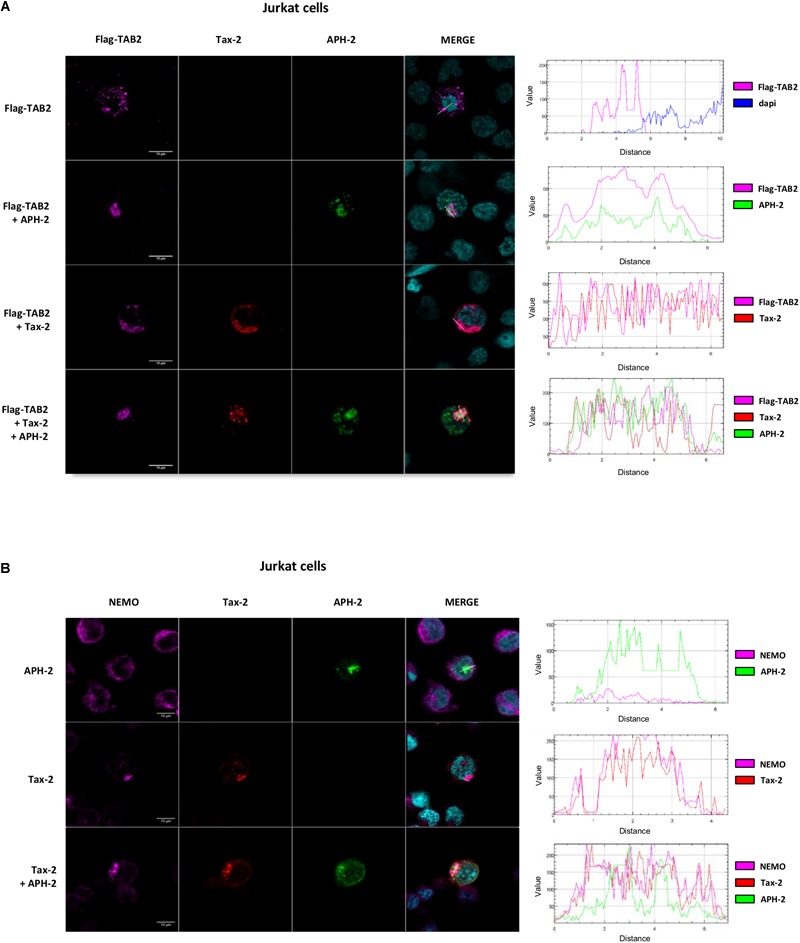
APH-2 is present in cytoplasmic complexes with TAB2, NEMO, and Tax-2. Jurkat cells were transfected with VSV-APH-2, Tax-2 and Flag-TAB2 expression vectors. Samples were stained with anti-VSV, anti-Tax-2, anti-Flag and anti-NEMO primary antibodies to detect APH-2 (green signal), Tax-2 (red signal), TAB2 (magenta signal), and NEMO (magenta signal), respectively **(A,B)**. Nuclei were stained with DAPI (cyan signal). The intensity of fluorescence overlapping the white line drawn on the merged images is plotted in the diagrams. Scalebar, 10 μm.

We have previously shown that Tax-2 co-localizes in the cytoplasm with the NF-κB pathway factors TAB2, NEMO and p65 (Avesani et al., 2010). Given that APH-2 co-localizes with Tax-2 in the cytoplasm, we speculated that APH-2 may be recruited in complexes containing these NF-κB factors. To test this hypothesis, the co-localization of APH-2 with TAB2 and NEMO in Jurkat cells was analyzed by confocal microscopy (Figure 4). The results showed a redistribution of TAB2 in cytoplasmic structures in the presence of Tax-2 and APH-2. TAB2 partially co-localized with APH-2 in the absence of Tax-2 (Figure 4A). APH-2 co-localized with NEMO only in the presence of Tax-2 (Figure 4B). The co-localization of Tax-2 and APH-2 with NEMO was also reproduced in U2OS cells (Supplementary Figure 4A). Taken together, these results suggest that in the presence of Tax-2, APH-2 localized in the cytoplasm with TAB2 and NEMO.

### HBZ and APH-2 Interfere With TRAF3 Functions

**FIGURE 5:**
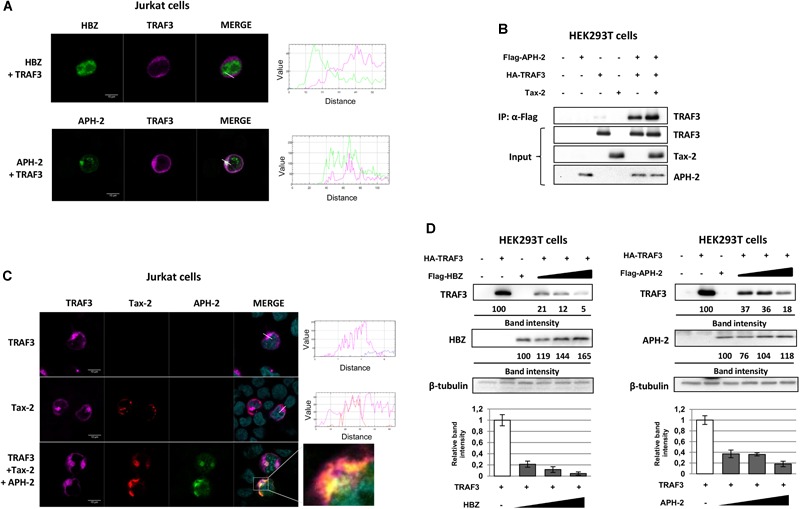
HBZ and APH-2 interfere with TRAF3 functions. **(A)** Jurkat cells were transfected with Flag-HBZ or VSV-APH-2 expression vectors. Samples were stained with an anti-TRAF3 and anti-Flag or anti-VSV primary antibodies to detect TRAF3 (magenta signal) and HBZ or APH-2 (green signal), respectively. **(B)** HEK293T cells were transfected with HA-TRAF3, Tax-2 and Flag-APH-2 expression vectors. Tagged proteins were immunoprecipitated with an anti-Flag antibody and the presence of the TRAF3 protein was examined with an anti-HA primary antibody, by western blot. **(C)** Jurkat cells were transfected with VSV-APH-2 and Tax-2 expression vectors. Samples were stained with anti-VSV, anti-Tax-2 and anti-HA primary antibodies to detect APH-2 (green signal), Tax-2 (red signal), and TRAF3 (magenta signal), respectively. Nuclei were stained with DAPI (cyan signal). The intensity of fluorescence overlapping the white line drawn on the merged images is plotted in the diagrams. Enlargements are shown bottom the “Merge” panel. Scale bar, 10 μm. **(D)** HEK293T were co-transfected with HA-TRAF3 and increasing amount of Flag-HBZ or Flag-APH-2. Immunoblot analysis (top) was performed and the amount of TRAF3, HBZ and APH-2 was measured relative to the amount of β-tubulin (bottom).

Since the antisense proteins HBZ and APH-2 both repress NF-κB activity, we investigated the possible recruitment of HBZ and APH-2 in cytoplasmic complexes containing TRAF3. We analyzed TRAF3 distribution in the presence of viral antisense proteins by confocal microscopy in Jurkat cells. We found that HBZ (green signal) and TRAF3 (magenta signal) did not co-localize, whereas TRAF3 partially co-localized with APH-2 in the cytoplasm (Figure 5A). We confirmed that TRAF3 is present in immunocomplexes containing APH-2 by co-immunoprecipitation (Figure 5B). Furthermore, we detected TRAF3 in complexes containing both APH-2 and Tax-2 (Figure 5B). We analyzed by confocal microscopy TRAF3 distribution in the presence of APH-2 and Tax-2, and we found that APH-2 (green signal), TRAF3 (magenta signal) and Tax-2 (red signal) co-localized in the cytoplasm (Figure 5C). Co-localization of Tax-2 and APH-2 with TRAF3 was also observed in U2OS cells (Supplementary Figure 4B). This indicates that APH-2, but not HBZ, could modulate Tax-2 induced NF-κB activation by associating with Tax-2 and TRAF3 complexes.

To assess whether HBZ may deregulate the NF-κB pathway by affecting the expression of TRAF3, as it was previously shown with p65, HEK293T cells were transfected with increasing amounts of HBZ and the expression levels of TRAF3 was evaluated. The results demonstrated a reduction of the expression of TRAF3 protein in the presence of HBZ and APH-2 (Figure 5D). A reduced expression of endogenous TRAF3 was also found in the presence of increasing amounts of HBZ and APH-2 (Supplementary Figures 5A,B). These results suggest that TRAF3 may be involved in the mechanisms of NF-κB deregulation mediated by HTLV proteins and that its expression may be altered by both HBZ and APH-2 proteins.

## Discussion

Dysregulation of the NF-κB pathway can induce alterations of physiological cellular responses and can lead to oncogenesis (Park and Hong, 2016). It has been demonstrated that persistent NF-κB activation derived from Tax expression induces cell senescence, and the inhibitory effect of the antisense protein HBZ on NF-κB may represent an adaptation of HTLV-1 that contributes to viral persistence and, in the long term, to ATL development (Giam and Semmes, 2016). It has also been reported that the inhibition of the NF-κB pathway results in the induction of apoptosis of ATL cells (Watanabe et al., 2005). The HTLV regulatory proteins interaction with canonical NF-κB factors leading to NF-κB dysregulation has been intensively investigated, but limited information is available about the interaction with factors that play a critical role in the non-canonical pathway (Schmitz et al., 2014; Fochi et al., 2018). We focused our attention on TRAF3, a ubiquitin ligase that controls the expression levels of the NIK kinase and that has been demonstrated to be abnormally expressed in freshly isolated ATL cells (Watanabe, 2017). TRAF3-deficient mice display increased non-canonical NF-κB activation as well as increased B cell survival, suggesting that TRAF3 may act as a negative regulator of the NF-κB signaling *in vivo* (Gardam et al., 2008). It has been demonstrated that TRAF3 knockout results in NIK stabilization, inducing p100 processing (Liao et al., 2004; He et al., 2006). In TRAF3-deficient cell lines, we found that the NF-κB pathway is activated and that both a partial nuclear distribution of the transcriptional factor p65 and p100 processing are taking place. These results confirm that TRAF3 is a negative regulator of the NF-κB pathway, but it may also participate to the functional regulation between the two NF-κB pathways. We also found that p65 and TNF-α partially induced NF-κB activation in TRAF3-KO cell lines. This effect may be explained by negative feedback loop mechanisms derived by the expression of NF-κB target genes acting as negative regulators. This hypothesis is supported by the qRT-PCR analyses of A20 and BCL-2 genes in TRAF3-KO cells, showing that they are over-expressed as compared to WT cells. It is known that A20 mediates a negative regulation of the canonical NF-κB pathway (Pujari et al., 2013) and BCL-2 overexpression specifically represses NF-κB-dependent transactivation by attenuating the transactivation potential of p65 (Grimm et al., 1996). These results are consistent with previous data showing that TRAF3 depletion causes an accumulation of distinct subsets of NF-κB inhibitors, including A20 (Bista et al., 2010).

We have previously shown that Tax interacts with TRAF3 and here we demonstrate for the first time that the TRAF3 factor is required for an efficient NF-κB activation by both Tax-1 and Tax-2.

Unlike Tax-1, Tax-2 cannot induce p100 processing to p52, but can activate the canonical NF-κB pathway as well as Tax-1 (Xiao et al., 2001; Higuchi et al., 2007). We found that Tax-mediated activation of NF-κB is impaired in TRAF3-deficient cells and that it is restored only after rescuing TRAF3 expression. A similar essential role of TRAF3 has been demonstrated for the Epstein-Barr virus-encoded oncoprotein latent membrane protein 1 (LMP1). In fact, LMP1 alone can transform rodent fibroblasts and activate NF-κB (Cahir McFarland et al., 1999), but in the absence of TRAF3, LMP1-induced activation of JNK, p38, and NF-κB are impaired (Xie et al., 2004). It has been also demonstrated that the vFlip protein expressed by Kaposi’s sarcoma-associated herpesvirus (KSHV/HHV8) activates NF-κB interacting with the IKK complex and the TRAF2/3 complex (Field et al., 2003). Interestingly, we found that TRAF3 is required not only for Tax-1, but also for Tax-2 NF-κB activation, supporting a mechanism in which TRAF3 may be involved in the association with factors that cooperate in the canonical and non-canonical NF-κB pathway.

We report here a reduction of TRAF3 expression in the presence of the antisense proteins. We would have expected an activation of the non-canonical NF-κB pathway, but we have not observed an accumulation of p52 in the presence of HBZ (data not shown). This result is in agreement with data demonstrating a lowered expression of p100 in the presence of HBZ that leads to lowered amounts of the non-canonical transcription factor p52 (Zhi et al., 2011). The HBZ effect in the non-canonical NF-κB activation still remains controversial since Zhao et al. (2009) suggest that HBZ inhibits selectively the canonical NF-κB pathway in the presence of Tax-1.

Zhao et al. (2009) and Panfil et al. (2016) demonstrated that both HBZ and APH-2 interact with p65 and reduce p65-mediated NF-κB promoter activity. We show here that HBZ and APH-2 may exert a different molecular mechanism in the down-modulation of the NF-κB pathway. We found that HBZ is less efficient in dampening Tax-mediated NF-κB activity compared to APH-2 and that co-expression of HBZ and high levels of Tax-1 results in restoring NF-κB activation, whereas APH-2 maintains its inhibitory effect despite high expression levels of Tax-2. We also observed that APH-2 inhibits p65-induced NF-κB activation in both HEK293T and Jurkat cell models. This is in contrast to the previous report showing that APH-2 inhibits the p65-induced NF-κB activation only in HEK293T cells (Panfil et al., 2016). This discrepancy may be due to differences in the efficiency of cell transfection, as declared by the authors.

**FIGURE 6:**
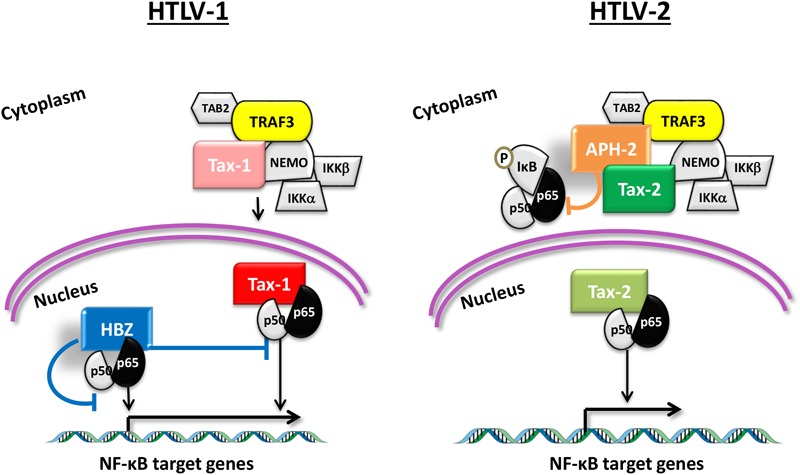
Molecular model for HTLV-1 and HTLV-2 regulatory proteins HBZ and APH-2 on Tax-mediated NF-κB activation and TRAF3 interaction. In the presence of Tax-1, HBZ inhibits p65 action in the nucleus competing with Tax-1. The recruitment of APH-2 in Tax-2-cytoplasmic complexes containing TRAF3 limits the p65 nuclear translocation.

By analyzing the subcellular distribution of the protein complexes formed by Tax and the host interacting proteins, we observed that APH-2, but not HBZ, is recruited in the cytoplasmic structures containing NEMO and TAB2. We have also demonstrated that the co-expression of Tax-2 and APH-2 results in the accumulation of the NF-κB inhibitor IκB and in a partial impairment of p65 nuclear translocation, whereas IκB expression is reduced when Tax-1 and HBZ are co-expressed. From the results obtained, we propose a model in which the recruitment of HBZ and APH-2 within the protein complexes formed by Tax follows different patterns. While APH-2 is partially retained in the cytoplasmic compartments, HBZ translocates more efficiently into the nucleus, thus inhibiting the transcription factor activity of p65 (Figure 6). This model is also supported by the results of Marban et al. (2012) that demonstrate a redistribution of APH-2 in the cytoplasm in the presence of Tax-2, preventing APH-2 from activating AP-1 transcription.

Our results clearly demonstrate that TRAF3 is required for NF-κB dysregulation mediated by Tax, thus representing a novel factor recruited by the viral regulatory proteins to alter cell pathways. In fact, TRAF3 not only acts on NF-κB pathway, but also plays significant roles in the immunity-related signal transduction (Häcker et al., 2011). Cells lacking TRAF3 are defective in IFN-I responses activated by different TLRs (Oganesyan et al., 2006). The induction of IFN-I elicits the activation of the antiviral cellular gene expression that coincides with the inhibition of viral replication. The key role of TRAF3 in promoting antiviral signaling is supported by a recent study demonstrating that the deletion of TRAF3 in adult mice attenuated their host defense against vesicular stomatitis virus (VSV) infection (Xie et al., 2019). In the non-canonical NF-κB pathway, TRAF3, in concert with TRAF2, cIAP1 and cIAP2, promotes the constitutive degradation of NIK that in turn serves as negative regulator of IFN (Jin et al., 2014; Parvatiyar et al., 2018). A feedback inhibition mechanism has been also proposed to induce TRAF3 degradation, affecting IFN production and NIK accumulation (Nakhaei et al., 2009). Recent evidence demonstrates that the HTLV antisense proteins interact with IRF-1, a transcriptional regulator of IFN-I pathway. In particular, APH-2 enhances IRF-1 DNA binding and steady-state expression levels, whereas HBZ interacts with IRF-1 and causes its degradation (Panfil et al., 2016). The loss of IRF-1 expression has been observed in several cases of leukemia (Alsamman and El-Masry, 2018). It is reasonable to think that the recruitment of APH-2 and Tax-2 in complexes with TRAF3 may positively affect IFN response. This hypothesis could be linked to the demonstration that in rabbits, the lack of APH-2 increases the infection rate of HTLV-2 mutants in comparison to HTLV-2 WT (Yin et al., 2012). Further studies will be required to analyze the interplay of TRAF3 and the HTLV antisense proteins in deregulating the type I IFN induction.

## Conclusion

In summary, we demonstrated that TRAF3 cell factor is required for Tax-mediated NF-κB activation. Our findings allow to further understand the roles of host factors that may be targeted by HTLV regulatory proteins and participate in the crosstalk between IFN and NF-κB pathway. We also found that APH-2 is more effective than HBZ in the inhibition of Tax-mediated NF-κB activation. We suggest that the recruitment of APH-2 in Tax-2-cytoplasmic structures with NF-κB factors that affect the p65 nuclear translocation may explain the different effects of APH-2 and HBZ. This work allows to shed light on the role of HTLV-induced NF-κB pathway activation and ultimately in the identification of potential therapeutic targets.

## Data Availability

The datasets generated for this study are available on request to the corresponding author.

## Author Contributions

SF, MS, SM, and EB performed the study and contributed to manuscript discussion. SF contributed to manuscript drafting and writing. RM, CJ, VC, DZ, and UB provided intellectual input and contributed to manuscript drafting. MR conceived and designed the experiments, coordinated the study, and contributed to manuscript drafting and writing.

## Conflict of Interest Statement

The authors declare that the research was conducted in the absence of any commercial or financial relationships that could be construed as a potential conflict of interest.

## Footnotes

1 http://crispr.mit.edu

2 http://chopchop.cbu.uib.no/
