# Supplementary figures and images for: TRAF3 Is Required for NF-κB Pathway Activation Mediated by HTLV Tax Proteins
Source: Front Microbiol. 2019 Jun 12;10:1302. doi: 10.3389/fmicb.2019.01302 (PMC6581700; doi:10.3389/fmicb.2019.01302)

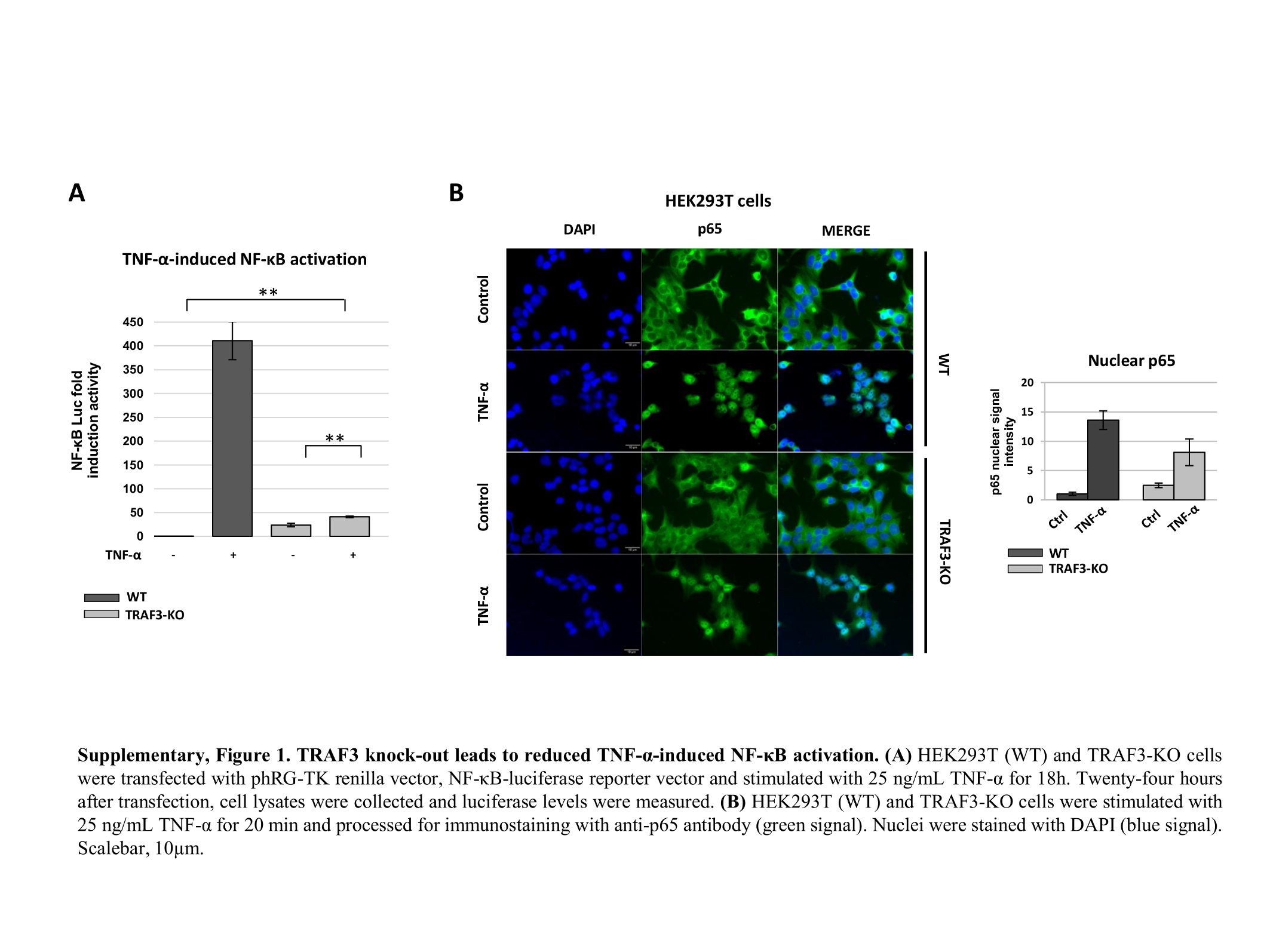

Supplement: Supplementary file 1 [file Image_1.TIF]

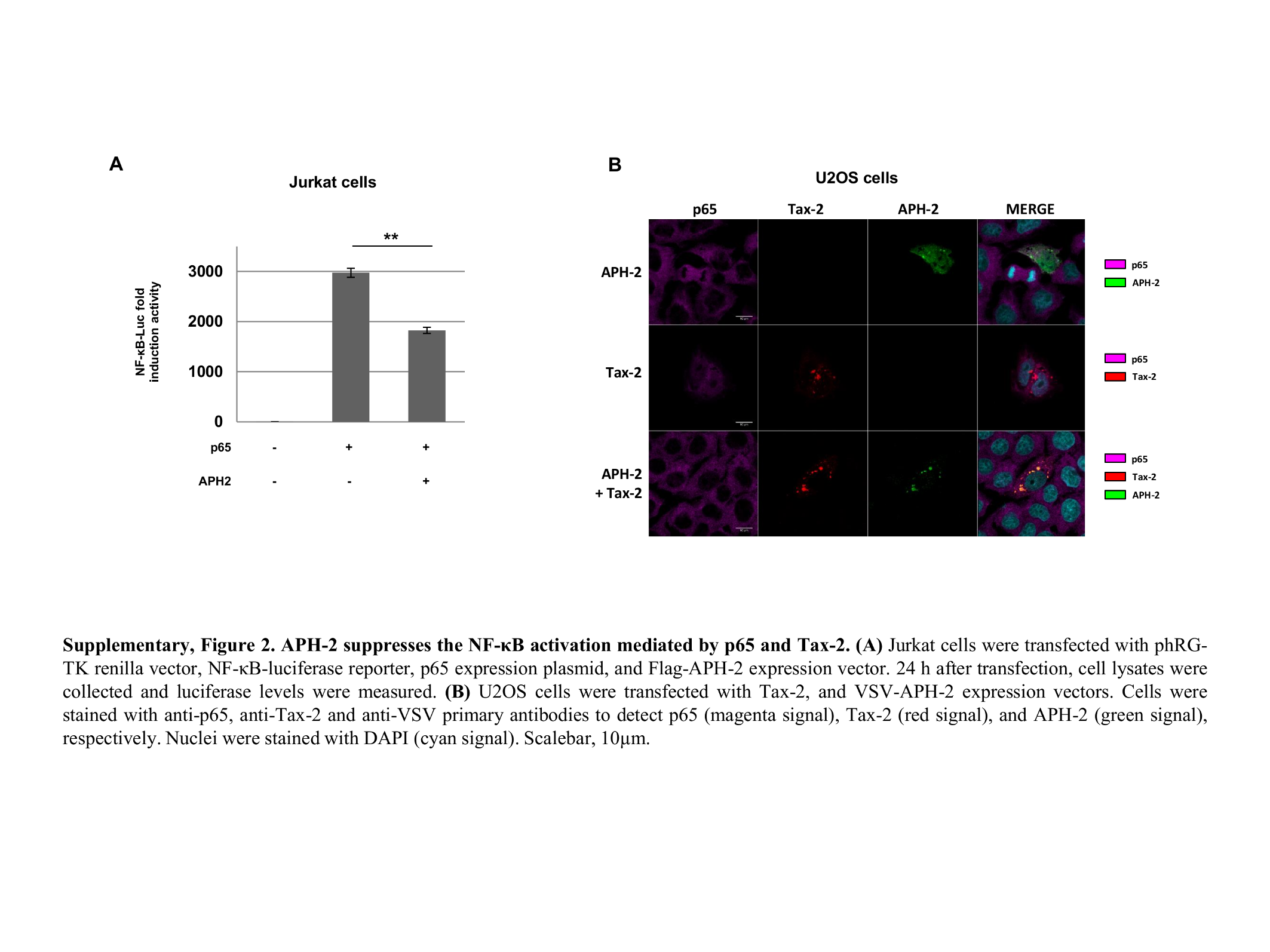

Supplement: Supplementary file 2 [file Image_2.TIF]

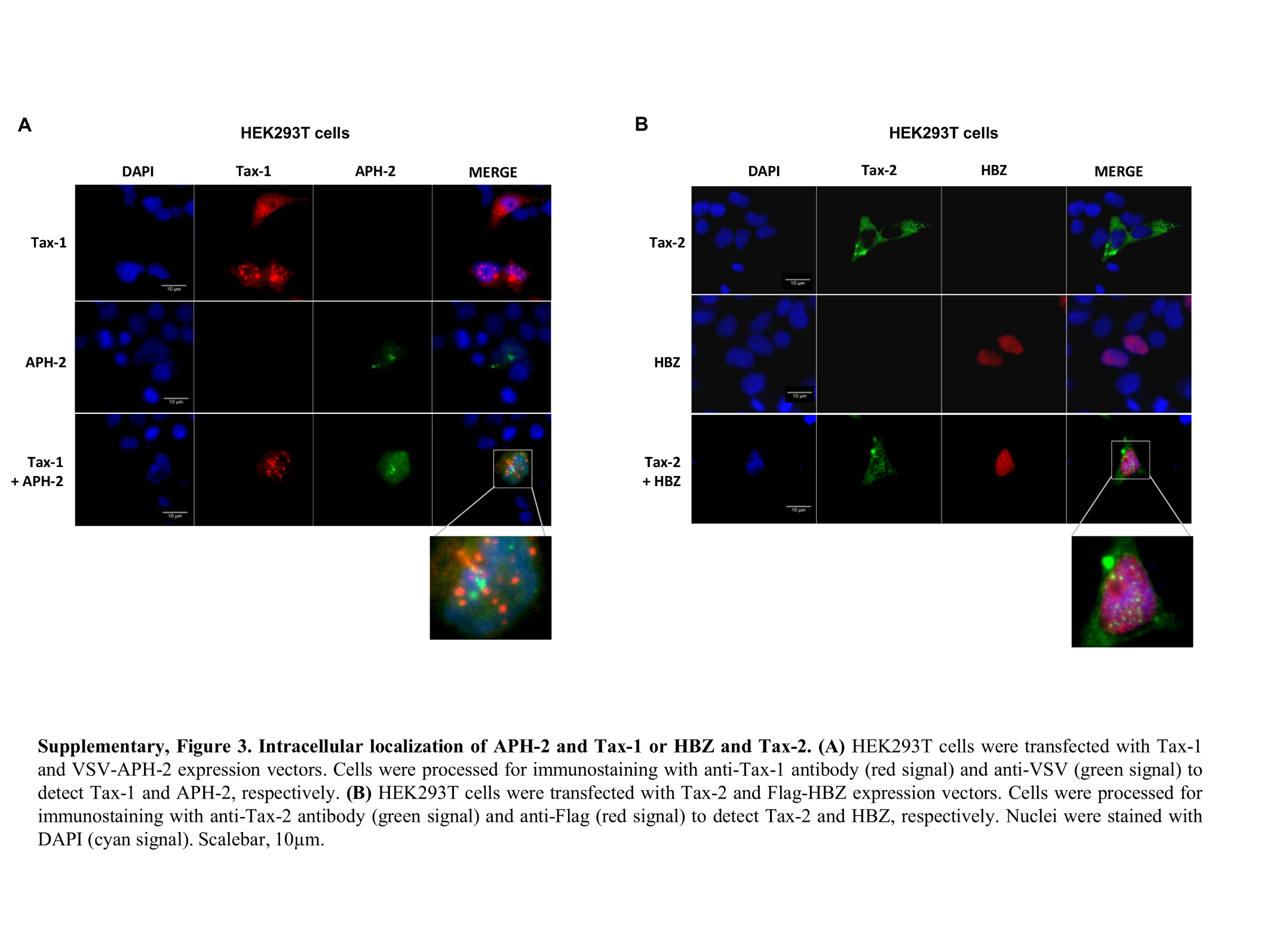

Supplement: Supplementary file 3 [file Image_3.TIF]

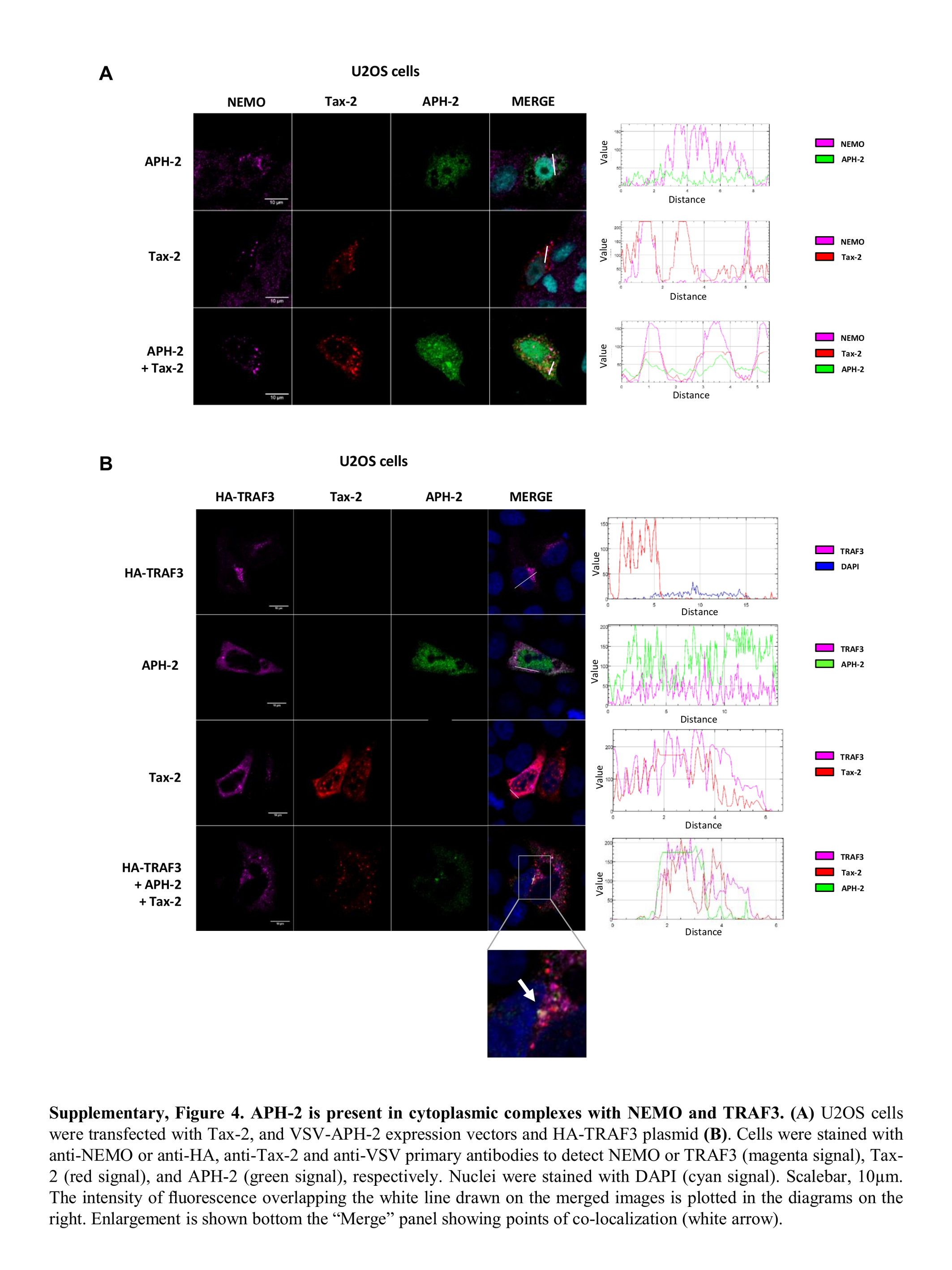

Supplement: Supplementary file 4 [file Image_4.TIF]

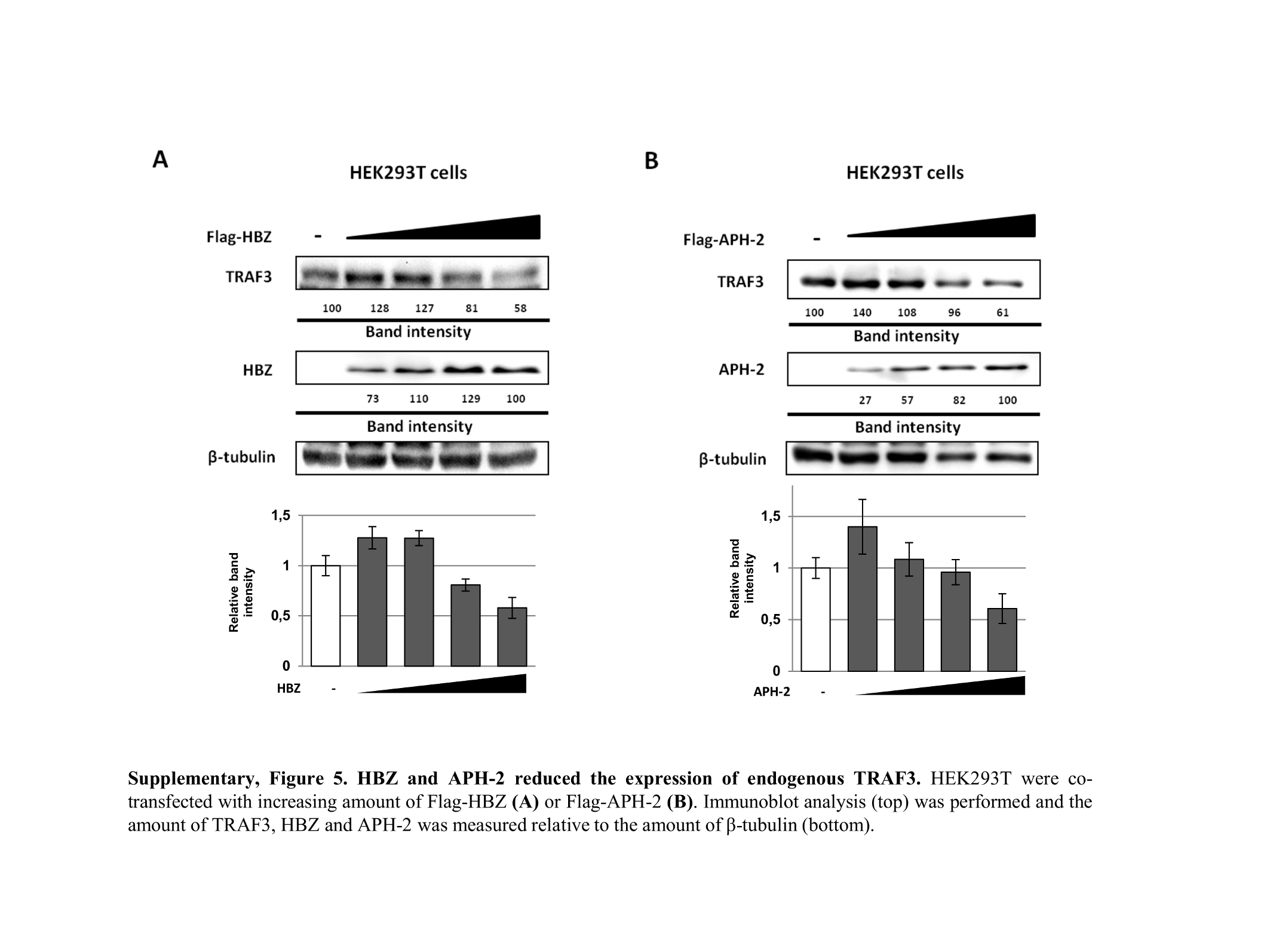

Supplement: Supplementary file 5 [file Image_5.TIF]
